# Factors associated with language difficulties and special educational needs among children with language difficulties: a retrospective cohort study

**DOI:** 10.1136/bmjpo-2025-004098

**Published:** 2026-10-26

**Authors:** Ertugrul Sancak, Ayse Mete Yesil, Emel Omercioglu, Buse Şencan Karakuş, Gökçenur Özdemir, Elif Nursel Özmert

**Affiliations:** 1 Department of Pediatrics, Hacettepe University Faculty of Medicine, Ankara, Turkey; 2 Division of Developmental Pediatrics, Department of Pediatrics, Hacettepe University Faculty of Medicine, Ankara, Turkey

**Keywords:** Health Policy, Child Health, Low and Middle Income Countries, Rehabilitation, Developing Countries

## Abstract

**Background:**

Language difficulties are a prevalent developmental concern, but prognostic heterogeneity makes early identification of high-risk children challenging. This study aimed to investigate factors associated with language difficulties and to identify early predictors of special educational needs (SEN) in Turkish-speaking children.

**Methods:**

A retrospective cohort study was conducted at a tertiary paediatric hospital in Türkiye. The analytical cohort for the analysis of factors associated with language difficulties consisted of 255 children with language difficulties and 51 children who were judged to have typical development after evaluation, all initially referred for speech delay between May 2014 and May 2021. Multivariable logistic regression was used to identify independent associations.

**Results:**

In the multivariable model, later age at presentation (adjusted OR 1.08 per month, 95% CI 1.01 to 1.16) and vitamin D deficiency (adjusted OR 4.22, 95% CI 1.00 to 17.75) were significantly associated with language difficulties. Within the language difficulties cohort (n=255), children with SEN (n=88) presented at a later age (mean difference 8.6 months, 95% CI 5.5 to 11.6) and had higher odds of screen exposure >2 hours/day (OR 2.1, 95% CI 1.1 to 4.2) compared with those without SEN (n=154).

**Conclusions:**

Later age at presentation and vitamin D deficiency were associated with language difficulties, while later age at presentation and increased screen exposure were associated with SEN among children with language difficulties. These findings highlight the importance of early screening and suggest that modifiable factors like screen time may be valuable intervention targets. The role of vitamin D in language disorders requires further prospective investigation.

**WHAT IS ALREADY KNOWN ON THIS TOPIC**

- Established risk factors for language difficulties include male gender, low parental education and socioeconomic status, family history and perinatal adverse events. Prognosis is highly heterogeneous, making early identification of high-risk children a priority.

**WHAT THIS STUDY ADDS**

- In Turkish-speaking children presenting with speech delay, later age at presentation was associated with language difficulties and with subsequent special educational needs.
- Screen exposure >2 hours/day was more prevalent in children with language difficulties and in those who subsequently required special education.
- Vitamin D deficiency was more common in children with language difficulties; further research is needed to clarify the nature of this association.

**HOW THIS STUDY MIGHT AFFECT RESEARCH, PRACTICE OR POLICY**

- At the policy level, the findings support earlier identification pathways that combine developmental screening with access to comprehensive developmental evaluation. These results also reinforce the importance of counselling families about modifiable environmental factors such as screen exposure.

## Introduction

 Language difficulties are among the most prevalent developmental concerns in early childhood. Within this broad clinical area, developmental language disorder (DLD) affects approximately 1 in 14 individuals.^1^ It is clinically characterised by difficulties in receptive and/or expressive language in any linguistic domain (morphology, syntax, semantics, pragmatics), for example, limited vocabulary and impaired sentence structure.^2 3^ The Diagnostic and Statistical Manual of Mental Disorders, Fifth Edition (DSM-5) provides diagnostic criteria for language disorder but omits severity gradations or subtypes.^4^

The terminology used for early language difficulties is heterogeneous across the literature, with terms such as isolated language disorder/delay, DLD, late talkers and specific language impairment (SLI) used in different clinical and research contexts.^5 6^ Although there is no complete terminological consensus in this field, these distinctions are often shaped by the clinical characteristics of the populations studied, accompanying findings and developmental course. In DSM-5, persistent language difficulties arising in the early developmental period are classified under language disorder, although the system does not define prognostic subgroups within this category. To reduce terminological inconsistency, the multinational and multidisciplinary CATALISE consensus recommended against the continued use of terms such as SLI and language delay, and proposed the term DLD for children over 5 years of age whose language difficulties are persistent and have a functional impact on daily life.^7^ We used the term language difficulties as a broader umbrella term for children with primary language difficulty identified on developmental assessment in the absence of significant motor, sensory or cognitive impairment. The prognosis within this group is quite heterogeneous. While most late talkers achieve age-appropriate language skills by preschool age,^2^ DLD is associated with persistent and lifelong difficulties.^1^ Longitudinal studies reveal that adults with childhood DLD tend to have lower socioeconomic status and higher unemployment rates compared with the general population.^8 9^ This heterogeneity necessitates tailored intervention strategies. Watchful waiting in cases with a favourable prognosis may prevent unnecessary burden on families and the healthcare system.^5^ Conversely, inadequate or delayed intervention in high-risk cases can lead to entrenched deficits and poorer long-term outcomes.^10^ In deciding on intervention strategies, it is extremely important to work in a multidisciplinary team that includes speech-language therapists/pathologists, paediatricians, developmental paediatricians, child development specialists and psychiatrists.

The marked prognostic heterogeneity underscores the critical need to identify high-risk subgroups requiring targeted intervention.

This retrospective cohort study aims to: (1) identify associated factors for language difficulties in Turkish-speaking children and (2) determine early predictors of the need for special education within this population. To achieve this, we first compared children with language difficulties to typically developing peers to identify factors associated with the disorder. Subsequently, within the language difficulties group, we compared children who developed a need for special education (either at presentation or during follow-up) to those who did not.

## Methods

This retrospective cohort study included patients presenting to Developmental Pediatrics Clinic at Hacettepe University İhsan Doğramacı Children’s Hospital between May 2014 and May 2021 with primary complaints of speech or language delay.

Collected variables included sociodemographic parameters (patients’ gender, age at evaluation), perinatal factors (gestational age, birth weight, neonatal intensive care unit admission, history of intubation, sepsis/meningitis, jaundice requiring phototherapy), familial factors (parental age and education, consanguinity, bilingualism, total number of children, primary caregiver, family history of speech/language delay), environmental exposures (screen time (duration/type/background exposure), frequency of literacy activities (shared book reading)), developmental markers (Denver II, Bayley Scales of Infant and Toddler Development, Third Edition (Bayley-III), Ages and Stages Questionnaire (ASQ) scores), laboratory investigations (complete blood count, vitamin D (25-OH vitamin D), vitamin B_12_, folate, metabolic/genetic testing when clinically indicated) and recommended intervention types (watchful waiting with language enrichment counselling, kindergarten attendance, special education).

Background television exposure was defined as television remaining audible and/or visible for >30 min while the child engaged in other activities in the same room, regardless of direct viewing.

Patient data were collected via structured developmental anamnesis interviews conducted with the primary caregivers, alongside standardised testing and electronic laboratory records.

Intervention recommendations were determined following a joint evaluation by a developmental paediatrician, a child development specialist and a clinical psychologist. Recommendations for special education were made based on longitudinal monitoring of the child’s language development. In addition, children with speech disorders or phonological errors, as well as some children with language difficulties requiring a special education report, were evaluated in collaboration with the Department of Speech and Language Therapy. In our country, after a special education report, insurance covers 2–3 hours of education per week. Special education takes place in private speech and language therapy centres under the control of the Ministry of Education.

Patients were evaluated every 3 months, and data collection points were established at these intervals.

All diagnoses adhered to DSM-5 criteria as follows^4^:

Language difficulties: Performance ≥2 SD below age norms in the language domain, in the absence of explanatory medical conditions.

Autism spectrum disorder (ASD): Met DSM-5 diagnostic criteria for social communication deficits and restricted, repetitive patterns of behaviour.

Global developmental delay (GDD): Significant delays (≥2 SD below the mean) in ≥2 developmental domains (gross/fine motor, cognition, language, personal-social).

Speech sound disorder: Persistent phonological errors exceeding developmental expectations.

### Standardised assessment tools

1. Denver Developmental Screening Test II (DDST-II): Used for developmental screening. The Turkish version has established validity and reliability.^11^
2. ASQ: Evaluates development in children aged 3–72 months. The Turkish version has established validity and reliability.^12^
3. Bayley-III: Comprehensively assesses development in children aged 1–42 months.^13^

Standardised developmental tests (Denver-II, ASQ and Bayley-III) were administered by Child Development Specialists who hold Master’s degrees in Child Development and are certified in the administration of standardised developmental tests.

### Assessment protocol

1. First-tier screening:
  - DDST-II.
  - ASQ (for children aged 3–72 months).
2. Second-tier evaluation:
  - Bayley-III (for children aged 1–42 months).
3. Diagnostic confirmation: Final diagnoses were established by a developmental paediatrician through comprehensive evaluation integrating standardised test results, direct clinical observations and detailed developmental history.

### Statistical analysis

Data were analysed using SPSS Statistics V.26.0 (IBM). The normality of continuous variables was assessed using the Kolmogorov-Smirnov test. Normally distributed continuous variables are reported as mean±SD and were compared using independent samples t-tests or one-way analysis of variance (ANOVA). Tukey HSD post hoc test was used following significant ANOVA results where equal variances were assumed. Non-normally distributed continuous variables are reported as median and IQR and were compared using the Mann-Whitney U test or Kruskal-Wallis H test. Categorical variables are presented as frequencies and percentages and were compared using the χ² test. Fisher’s exact test was used when >20% of cells had an expected count <5. Risk factor analysis was performed using univariable logistic regression. Variables with p<0.05 in univariable analyses were entered into a multivariable logistic regression model to identify independent predictors. A two-tailed p<0.05 was considered statistically significant.

## Results

This retrospective cohort study analysed 781 patients presenting with speech or language delay between May 2014 and May 2021. The final diagnostic distribution was as follows: language difficulties (n=322, 41.2%), GDD (n=158, 20.2%), ASD (n=126, 16.1%), speech sound disorder (n=65, 8.3%) and typical development (TD) (n=51, 6.5%). An additional 59 (7.6%) patients had bilateral sensorineural hearing loss and language delay.

Among the 322 language difficulties cases, 67 (20.8%) had comorbid medical conditions potentially influencing language development (eg, metabolic disorders, structural abnormalities, neurological conditions, other systemic diseases). After excluding these cases, final analytical cohort for language difficulties associated factor analysis comprised 255 children. The participant inclusion flow chart is presented in figure 1.

**Figure 1:**
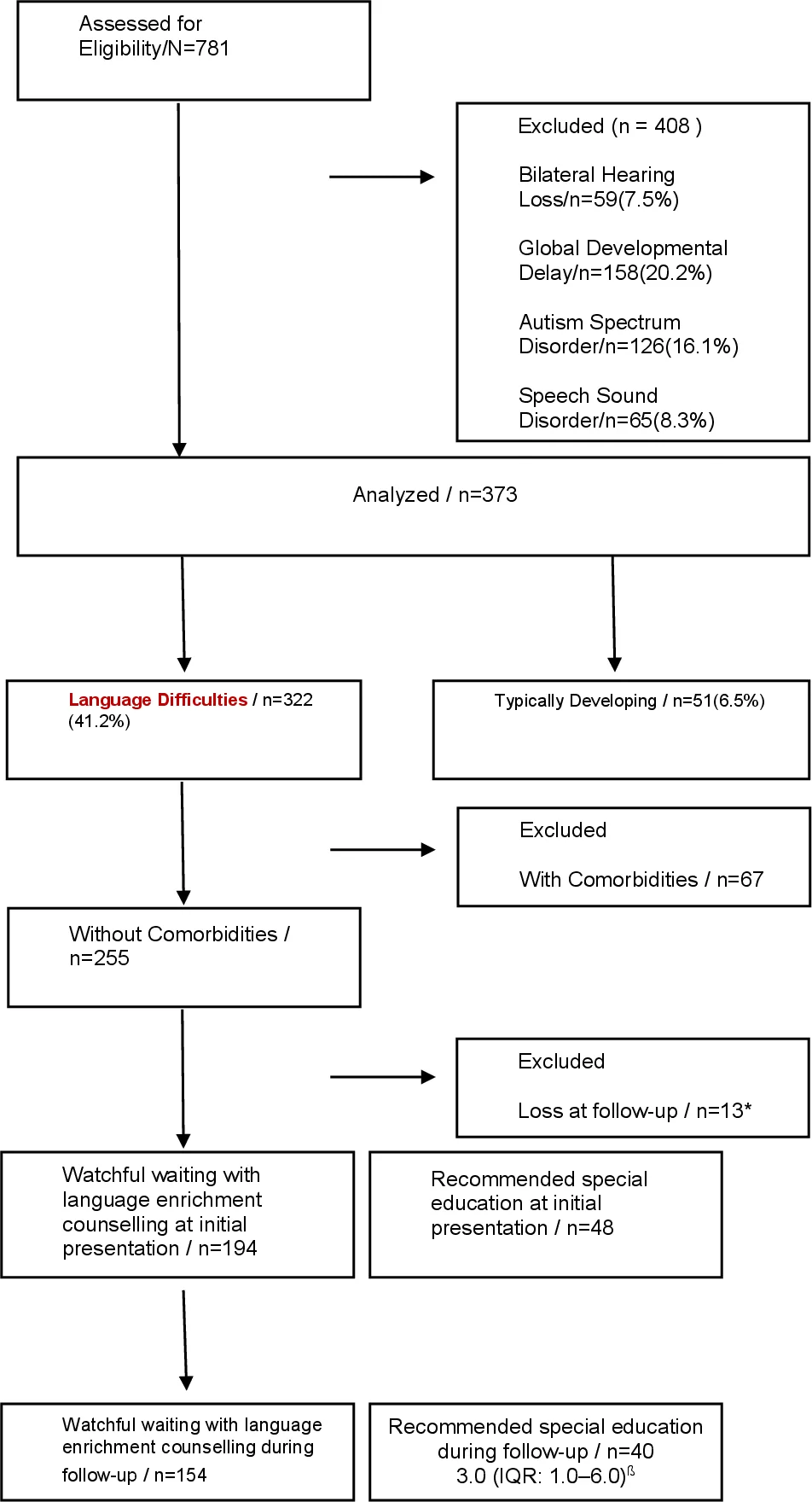
Participant inclusion flow chart. *13 lost to follow-up: four due to contact information unavailability and nine due to parental discontinuation of follow-up. ^ß^The median time from follow-up to recommendation for special education was 3.0 months (IQR 1.0–6.0).

### Associated factor analysis

To identify associated factors for language difficulties, the group of 255 children with language difficulties was compared with the group of 51 children evaluated as TD across sociodemographic, perinatal, familial and environmental characteristics (table 1).

**Table 1:** Comparison of clinical and demographic characteristics between children with language difficulties and typically developing children

	Language difficulties	Typically developing	P value
Total number of patients	255	51	
Gender			0.071
Male	187 (73.3%)	31 (60.8%)	
Female	68 (26.7%)	20 (39.2%)	
Age at admission (months) mean±SD	32.1±11.03	24.8±11.1	<0.001*
(Youngest-oldest)	(9–71)	(3–66)	
Maternal age (years) mean±SD	32.3±5.5	31.8±4.9	0.62
(Youngest-oldest)	(17–51)	(24–44)	
Mother education			
Illiterate	0	0	0.588
Primary school	26 (10%)	3 (6%)	0.26
Secondary school	23 (9%)	3 (6%)	0.543
High school	58 (23%)	12 (23.5%)	
University or higher	106 (41.5%)	22 (43%)	
Unknown	42 (16.5%)	11 (21.5%)	
Paternal age (years) Mean±SD	36.1±5.6	34.7±5.6	0.078
(Youngest-oldest)	(21–60)	(25–53)	
Father education			
Illiterate	0	0	0.775
Primary school	19 (7.5%)	4 (8%)	0.821
Secondary school	20 (8%)	3 (6%)	0.656
High school	56 (22%)	10 (19.5%)	
University or higher	110 (43%)	23 (45%)	
Unknown	50 (19.5%)	11 (21.5%)	
Consanguinity between parents			
Yes	35 (14%)	8 (15.5%)	0.747
They were born in the same village	18 (7%)	3 (6%)	
No	186 (73%)	37 (72.5%)	
Unknown	16 (6%)	3 (6%)	
Birth order			0.022*
1st child	131 (51.5%)	35 (68.5%)	
2nd child	79 (31%)	9 (17.5%)	
≥3rd child	34 (13.5%)	5 (10%)	
Unknown	11 (4%)	2 (4%)	
Total number of children in the family			0.003*
1	109 (43%)	33 (64.5%)	
2	93 (36%)	10 (19.5%)	
≥3	43 (17%)	6 (12%)	
Unknown	10 (4%)	2 (4%)	
Patients with a family member with speech delay	43 (17%)	4 (8%)	0.108
Bilingualism	4 (1.5%)	5 (10%)	0.008*
Who cares for the child			0.14
Mother and father	171 (67%)	31 (60.5%)	
Mother and relative (maternal/paternal grandmother, aunt, older sister)	41 (16%)	16 (31.5%)	
Orphanage	1 (0.5%)	0	
Mother and neighbour	2 (1%)	0	
Mother and caregiver	24 (9.5%)	3 (6%)	
Unknown	16 (6%)	1 (2%)	
Kindergarten attendance			0.033*
Going	51 (20%)	4 (8%)	0.601
Not going	193 (75.5%)	46 (90%)	
Playgroup	4 (1.5%)	0	
Unknown	7 (3%)	1 (2%)	
Shared book reading			0.621
Yes (everyday)	106 (41.5%)	24 (47%)	
Not regular	32 (12.5%)	3 (6%)	
No	92 (36%)	21 (41%)	
Unknown	25 (10%)	3 (6%)	
Screen time			0.03*
None	27 (10.5%)	11 (21.5%)	0.047*
0–60 min	72 (28%)	16 (31.5%)	
61–120 min	54 (21%)	11 (21.5%)	
121–180 min	30 (11.5%)	2 (4%)	
More than 180 min	61 (24%)	9 (17.5%)	
Unknown	11 (5%)	2 (4%)	
Background TV exposure			0.301
Open	158 (62%)	28 (55%)	
Closed	72 (28%)	18 (35%)	
Unknown	25 (10%)	5 (10%)	

* Are significant at the p<0.05 significance level.

TV, television.

Children in the language difficulties group presented at a significantly later age (mean=32.1±11.03 months) compared with the TD group (mean=24.8±11.1 months) (mean difference 7.2 months, 95% CI 3.9 to 10.5; t(304)=4.42, p<0.001).

Higher birth order (being the second or later child) was significantly associated with language difficulties (χ²(1, N=293)=5.23, p=0.022). Similarly, having two or more siblings was significantly more common in the language difficulties group (χ²(1, N=294)=8.66, p=0.003).

Bilingualism was significantly more prevalent in the TD group (p=0.008).

A significant difference was found in kindergarten attendance at the time of admission between the groups (20% vs 8%; χ²(1, N=294)=4.54, p=0.033). However, given the significant age difference at presentation between groups, this analysis was restricted to children over 36 months of age. In this age-matched subanalysis, the difference in kindergarten attendance was no longer significant (p=0.6).

Children with language difficulties were significantly more likely to be exposed to any amount of daily screen time compared with typically developing children (84.5% vs 74.5%; χ²(1, N=293)=4.68, p=0.030). Furthermore, when examining excessive screen use, exposure exceeding 2 hours/day was also significantly more prevalent in the language difficulties group (35.5% vs 21.5%; χ²(1, N=293)=3.96, p=0.047).

Laboratory evaluations are displayed in table 2. Vitamin D deficiency was significantly more common in the language difficulties group compared with the TD group (59% vs 18%; χ²(1, N=112)=9.86, p=0.002). Given that screen exposure was also higher in the language difficulties group, we investigated whether the observed vitamin D deficiency could be a secondary effect of increased indoor activity. However, subsequent analysis found no significant association between vitamin D deficiency (<20 ng/mL) and various screen time thresholds in the sample tested (>2 hours/day, χ²(1)=1.196, p=0.274), suggesting that these may be independent associated factors.

**Table 2:** Comparison of laboratory values between children with language difficulties and typically developing children

	Language difficulties	Typically developing	P value
Total number of patients	255	51	
Haemoglobin	N: 193	N: 36	1
Less than 110 g/L (%)*	14 (7.2%)	2 (5.5%)	
Ferritin	N: 169	N: 29	0.713
Below 15 ng/mL (%)*	87 (51.4%)	16 (55%)	
B_12_ deficiency	N: 161	N: 25	0.784
<200 ng/L (%)*	29 (18%)	5 (20%)	
200–300 ng/L (%)*	52 (32.5%)	10 (40%)	
>300 ng/L (%)*	80 (49.5%)	10 (40%)	
Folic acid deficiency	N: 82	N: 7	
<4 ng/mL (%)*	1 (1.2%)	0	Could not be evaluated
>4 ng/mL (%)*	81 (98.8%)	7 (100%)	
TFT	N: 172	N: 25	
Hypothyroidism (high TSH, low or normal free T4 according to patient age) (%)*	6 (3.5%)†	0	Could not be evaluated
Hyperthyroidism (low TSH, high or normal free T4 according to patient age) (%)*	8 (4.5%)†	0	
25-OH vitamin D	N:95	N:17	0.002‡
<12 ng/mL (%)*	23 (24.3%)	1 (6%)	
12–20 ng/mL (%)*	33 (34.7%)	2 (12%)	
>20 ng/mL (%)*	39 (41%)	14 (82%)	

* Percentage of pathological ones in the number of patients evaluated (N) is given.

† None of these patients were diagnosed with thyroid disease. After repeated testing, TFTs were consistently within the normal range.

‡ Are significant at the p<0.05 significance level.

TFT, thyroid function test; TSH, thyroid stimuation hormone.

### Predictive modelling for language difficulties

A multivariable logistic regression model was constructed to identify independent factors associated with language difficulties. Variables that showed a significant association (p<0.05) in univariable analyses were included in the model (Kindergarten attendance lost its significance when adjusted for the 36-month age limit for kindergarten in our country and was therefore excluded. Bilingualism was also excluded due to the very low number of individuals in the groups (4 and 5 children). Birth order and number of siblings were highly collinear; therefore, only number of siblings, which showed the stronger univariable association, was retained in the final model. The overall model was statistically significant (model χ²=17.15, p=0.002) and demonstrated good fit according to the Hosmer-Lemeshow goodness-of-fit test (p=0.436). The model correctly classified 85.4% of all cases, with a sensitivity of 97.7% for language difficulties and a specificity of 23.5% for TD.

In the multivariable model, later age at presentation and vitamin D deficiency remained significant independent predictors. For each 1-month increase in age at presentation, the odds of having language difficulties increased by 8% (adjusted OR=1.08; 95% CI 1.006 to 1.158; p=0.032). Children with vitamin D deficiency had 4.22 times higher odds of being diagnosed with language difficulties compared with those with sufficient levels (adjusted OR=4.22; 95% CI 1.003 to 17.746; p=0.049). The results of the full multivariable logistic regression analysis are presented in table 3.

**Table 3:** Multivariable logistic regression analysis of associated factors for language difficulties

	Adjusted OR	95% CI	P value
Age at admission (per month)	1.080	1.006 to 1.158	0.032*
Vitamin D deficiency	4.219	1.003 to 17.746	0.049*
Number of siblings	2.084	0.596 to 7.283	0.250
Screen exposure	0.653	0.135 to 3.156	0.596

* Are significant at the p<0.05 significance level.

### Predictors of special education needs in language difficulties cohort

Children with language difficulties (n=255) were categorised based on their intervention trajectory: (1) recommended watchful waiting with language enrichment counselling (n=154), (2) recommended special education intervention on initial presentation (n=48) and (3) recommended special education intervention during follow-up (n=40). The median time (month) elapsed between follow-up and recommendation for special education was 3.0 (IQR: 1.0–6.0). 13 children from the original language difficulties cohort were excluded from this analysis due to loss at follow-up. The characteristics of these groups are compared in table 4.

**Table 4:** The characteristics of children with language difficulties by intervention trajectory

	Watchful waiting with language enrichment counselling	Recommended special education during follow-up	Recommended special education at initial presentation	P value
Total number of patients	154	40	48	
Gender				0.197
Male	106 (69%)	32 (80%)	38 (79%)	
Female	48 (31%)	8 (20%)	10 (21%)	
Age at admission (month)				
Mean±SD	28.6±8.6	32.4±9.5	41.3±13.5	<0.001*
(Youngest-oldest)	(9–57)	(13–64)	(17–71)	
Maternal age (year) Mean±SD	32.2±5.9	32.5±4.9	32.2±5.1	0.934
(Youngest-oldest)	(17–51)	(19–41)	(24–44)	
Maternal education				0.896
Illiterate	0	0	0	0.868
Primary school	14 (9%)	3 (7.5%)	5 (10.5%)	0.812
Secondary school	13 (8.5%)	5 (12.5%)	5 (10.5%)	
High school	35 (23%)	8 (20%)	11 (23%)	
University or higher	68 (44%)	17 (42.5%)	19 (39.5%)	
Unknown	24 (15.5%)	7 (17.5%)	8 (16.5%)	
Maternal employment				0.609
Yes	47 (30.5%)	11 (27.5%)	11 (23%)	
No	93 (60.5%)	25 (62.5%)	32 (66.5%)	
Unknown	14 (9%)	4 (10%)	5 (10.5%)	
Paternal age (year) Mean±SD	36.2±5.9	36±5.5	36±4.9	0.994
(Youngest-oldest)	(21–60)	(26–46)	(25–50)	
Paternal education				0.741
Illiterate	0	0	0	0.907
Primary school	8 (5%)	2 (5%)	4 (8.25%)	0.689
Secondary school	12 (8%)	4 (10%)	3 (6.25%)	
High school	34 (22%)	10 (25%)	10 (21%)	
University or higher	70 (45.5%)	15 (37.5%)	23 (48%)	
Unknown	30 (19.5%)	9 (22.5%)	8 (16.5%)	
Consanguinity between parents				0.183
Yes	24 (15.5%)	6 (15%)	2 (4.2%)	
They were born in the same village	8 (5%)	1 (2.5%)	5 (10.4%)	
No	116 (75.5%)	31 (77.5%)	36 (75%)	
Unknown	6 (4%)	2 (5%)	5 (10.4%)	
Child’s birth order				0.116
1st child	83 (54%)	25 (62.5%)	18 (37.5%)	
2nd child	41 (26.5%)	12 (30%)	22 (45.5%)	
≥3rd child	26 (17%)	3 (7.5%)	4 (8.5%)	
Unknown	4 (2.5%)	0	4 (8.5%)	
Total number of children in the family				0.168
1	70 (45.5%)	20 (50%)	14 (29.25%)	
2	52 (34%)	17 (42.5%)	23 (48%)	
≥3	29 (18.5%)	3 (7.5%)	7 (14.5%)	
Unknown	3 (2%)	0	4 (8.25%)	
Have a family member with speech delay	23 (15%)	11 (27.5%)	8 (16.5%)	0.189
Bilingualism	4 (2.5%)	0	0	0.3
Who cares for the child				
Mother and father	105 (68.5%)	26 (65%)	31 (64.75%)	0.863
Mother and relative	26 (17%)	7 (17.5%)	7 (14.5%)	0.957
(maternal/paternal grandmother, aunt or older sister)				0.866
Orphanage	0	1 (2.5%)	0	
Mother and neighbour	0	1 (2.5%)	1 (%2)	
Mother and caregiver	18 (11.5%)	3 (7.5%)	3 (6.25%)	
Unknown	5 (3%)	2 (5%)	6 (12.5%)	
Kindergarten attendance				0.073
Going	27 (17.5%)	6 (15%)	13 (27%)	
Not going	124 (80.5%)	33 (82.5%)	30 (62.5%)	
Playgroup	2 (1.4%)	0	2 (4.25%)	
Unknown	1 (0.6%)	1 (2.5%)	3 (6.25%)	
Shared book reading				0.33
Yes (everyday)	70 (45.5%)	13 (32.5%)	17 (35.5%)	
Not regular	17 (11%)	6 (15%)	6 (12.5%)	
No	55 (35.5%)	18 (45%)	18 (37.5%)	
Unknown	12 (8%)	3 (7.5%)	7 (14.5%)	
Screen exposure time				0.029*
None	21 (13.5%)	3 (7.5%)	1 (2%)	
0–60 min	42 (27.5%)	11 (27.5%)	12 (25%)	
61–120 min	32 (21%)	8 (20%)	12 (25%)	
121–180 min	14 (9%)	9 (22.5%)	7 (14.5%)	
More than 180 min	40 (26%)	8 (20%)	12 (25%)	
Unknown	5 (3%)	1 (2.5%)	4 (8.5%)	
Background TV exposure				0.638
Open	95 (61.5%)	27 (67.5%)	30 (62.5%)	
Closed	47 (30.5%)	10 (25%)	11 (23%)	
Unknown	12 (8%)	3 (7.5%)	7 (14.5%)	

* Are significant at the p<0.05 significance level.

A one-way ANOVA revealed a significant difference in the age at presentation between the three groups (F(2, 241)=30.02, p<0.001). Post hoc analyses (Tukey HSD) indicated that children recommended special education at presentation were significantly older at presentation than both the watchful waiting group (p<0.001) and the group recommended special education during follow-up (p<0.001).

Due to low expected cell counts, the two groups requiring special education (at presentation and during follow-up) were combined for subsequent analyses concerning screen time. A χ² test of independence showed a significant association between the need for special education and daily screen exposure greater than 2 hours (χ²(1, N=232)=4.77, p=0.029). Screen exposure >2 hours was significantly more prevalent in children who required special education compared with those managed with watchful waiting alone.

## Discussion

This study provides a detailed analysis of the distribution and factors associated with speech and language delay in a large Turkish cohort, highlighting two key findings: later age at presentation as an important marker for language difficulties and subsequent need for special education, and higher screen exposure among children with language difficulties who subsequently required special education.

Among the 781 children presenting with primary speech/language concerns, nearly half (41.2%) were diagnosed with language difficulties, while a significant proportion had GDD (20.2%) or ASD (16.1%). This distribution underscores a key clinical implication: a complaint of speech delay necessitates a comprehensive developmental evaluation to rule out broader neurodevelopmental conditions and ensure appropriate intervention. Given the diversity of diagnostic outcomes in children presenting with speech and language concerns, multidisciplinary assessment involving speech and language therapists/pathologists, child and adolescent psychiatry and paediatric neurology is particularly important.

Our analysis identified several factors that were significantly more prevalent in the language difficulties group. Consistent with some previous studies, we found that later birth order and having two or more siblings were observed more frequently.^14^ This association may be mediated by reduced one-on-one caregiver interaction and language modelling in larger families, where parental attention and resources are divided. Furthermore, and aligning with current literature, screen exposure emerged as a significant factor.^15 16^ Both any screen exposure and exposure exceeding 2 hours per day were significantly more prevalent in the language difficulties group. This finding strongly supports the American Academy of Pediatrics (AAP) guidelines recommending no screen time before 18–24 months and limited, high-quality co-viewing thereafter. It is important to note that while these factors (birth order, number of siblings and screen exposure) were significant in univariable analyses, they were not retained as independent predictors in the final multivariable model.

The most salient findings from our predictive modelling are the independent roles of vitamin D deficiency and later age at presentation. After controlling for confounders, vitamin D deficiency was significantly more common in children with language difficulties and was associated with a 4.2-fold increase in the odds of the disorder. The biological plausibility of this relationship warrants further investigation, but may involve vitamin D’s role in neurodevelopment and neuroprotection. In addition, for each additional month in age at presentation, the odds of language difficulties increased by 8%. Later presentation may delay access to timely developmental evaluation and early intervention.

The potential mechanism linking vitamin D deficiency to language disorders remains unclear but may be rooted in its role in neurodevelopment. In adults, low vitamin D levels have been linked to impaired cognitive function.^17^ While one study found no association between childhood vitamin D levels and adolescent academic performance,^18^ other research aligns more closely with our findings. Several prenatal studies have reported correlations between low maternal vitamin D levels and poorer language outcomes in offspring,^19 20^ although interventional studies supplementing high-dose vitamin D during pregnancy have not demonstrated a protective effect.^21^ Most directly, a study from Japan found a significant association between vitamin D levels and language development in boys,^22^ lending cross-cultural support to our results. The discrepancy across studies highlights the need for further research to elucidate the nature and timing of vitamin D’s influence on language development.

The importance of early identification is underscored by our finding that later age at presentation was associated with both language difficulties and subsequent need for special education. This may reflect greater underlying difficulty at the time of referral, delayed access to developmental evaluation or both. AAP recommends broad developmental surveillance at 9, 18 and 30 months, with domain-specific screening initiated when concerns arise.^23^ Furthermore, field studies have highlighted the value of integrating structured developmental screening into health systems to improve the identification and management of early childhood developmental difficulties.^24^ Our findings support this staged approach and suggest that screening should be accompanied by access to comprehensive developmental evaluation and ongoing developmental monitoring. It is also noteworthy that our typically developing control group consisted of children initially referred for speech and language concerns. The fact that these children were judged to have TD after specialist assessment reinforces the value of expert developmental evaluation in triaging common parental concerns.

Within the language difficulties cohort, we sought to identify early markers of a more severe trajectory necessitating special education. Beyond well-established markers of poor prognosis, such as receptive language deficits and low socioeconomic status,^5^ our study points to two associated factors: higher screen exposure and later age at presentation. The strong association between later age at presentation and special education needs likely reflects accumulated deficits that are less responsive to brief counselling, thus requiring structured educational support. This prognostic heterogeneity within the language difficulties group, though not captured by DSM-5 criteria, is clinically paramount. Subgrouping patients based on age and risk factors, as explored in other studies,^25^ could optimise resource allocation and intervention planning. Because DSM-5 does not define prognostic subgroups within this category, this finding may partly reflect the inclusion of children with a transient ‘late talker’ trajectory within our broader language difficulties group.

Our study has several limitations. First, vitamin D levels were not seasonally adjusted, which may influence the prevalence of deficiency due to known fluctuations in synthesis. Second, the retrospective and cross-sectional design precludes the assessment of causal relationships and longitudinal language progression. Third, the composition of our control group (children referred for speech and language concerns but later deemed typically developing) may introduce a selection bias, potentially limiting the generalisability of the associated factor comparisons. Despite these limitations, this study represents the largest analysis of factors associated with language difficulties and special education needs in Turkish-speaking children to date.

In conclusion, our results underscore the dual role of later age at presentation as both a marker of language difficulties and an indicator of future need for specialised support. These findings support earlier comprehensive developmental evaluation and ongoing developmental monitoring in children presenting with speech delay. The observed association between screen time and poorer outcomes reinforces the importance of adhering to and promoting existing screen time guidelines. Our findings highlight an association between vitamin D deficiency and language difficulties that warrants further investigation. The clinical relevance of this finding remains to be determined and does not currently support routine vitamin D testing in this population. Future longitudinal studies with long-term follow-up are essential to validate these associations and explore language-specific prognostic markers.

## Data Availability

Data are available on reasonable request. All data relevant to the study are included in the article or uploaded as supplementary information.
