# Supplementary material for: Factors associated with language difficulties and special educational needs among children with language difficulties: a retrospective cohort study
Source: BMJ Paediatr Open. 2026 Oct 26;10(1):e004098. doi: 10.1136/bmjpo-2025-004098 (PMC13711527; doi:10.1136/bmjpo-2025-004098)
Supplement: Supplementary file 1 [file bmjpo-2025-004098.reviewer_comments.pdf]

PEER REVIEW HISTORY

BMJ Paediatrics Open publishes all reviews undertaken for accepted manuscripts. Reviewers are asked to complete a checklist review form and are provided with free text boxes to elaborate on their assessment. These free text comments are reproduced below.

ARTICLE DETAILS

**TITLE (PROVISIONAL)**	Factors associated with language difficulties and special educational needs among children with language difficulties: a retrospective cohort study
**AUTHORS**	Sancak, Ertugrul<br>Mete Yesil, Ayse<br>Omercioglu, Emel<br>Şencan Karakuş, Buse<br>Özdemir, Gökçenur<br>Özmert, Elif

VERSION 1 – REVIEW

**REVIEWER NAME**	pekcici, Bahar Bingöler
**REVIEWER AFFILIATION**	Ankara University, Department of Developmental Pediatrics
**REVIEWER CONFLICT OF INTEREST**	No Conflict of Interest
**AI DISCLOSURE**	No, I have not used AI
**DATE REVIEW RETURNED**	02-Feb-2026

**GENERAL COMMENTS**	My reviewer comments are as follows:<br>1- Title: As the study population consists exclusively of children diagnosed with isolated language disorder, the title should more clearly reflect ILD specifically. In addition, delayed diagnosis is reported as a predictor; but cannot be an predictor, rather be an associated factor, therefore, the wording of the title may need to be revised.<br>2- How this study might effect policy?- While the authors report diagnosing isolated language disorder, the policy implications of this study extend beyond language assessment alone. At the health system and policy level, the findings underscore the importance of comprehensive developmental evaluation, as focusing solely on language may overlook other developmental vulnerabilities that influence outcomes and service needs.<br>3- Introduction: The introduction would benefit from a more comprehensive background on ILD, including current knowledge and clinical considerations, before moving directly to the study aim.<br>4- Vitamin D and biological markers: The finding regarding vitamin D deficiency appears to be based on retrospective file data, and the number of available biological markers is very limited, which restricts meaningful analysis. Given this limitation, I would suggest that the manuscript focus more strongly on clinically actionable factors such as early diagnosis, early intervention, and reduction of excessive screen time. Consideration could be given to omitting vitamin D and other biological markers from the current manuscript. Prospective studies or randomized controlled trials would be needed for the role of vitamin D in children with ILD.<br>5- Terminology: The term “delayed diagnosis” would be more accurate than “delayed presentation.” It is well established that

	delayed diagnosis of ILD is associated with poorer outcomes and an increased need for special education services.<br>6- Screening and monitoring: In addition to screening, the importance of ongoing developmental monitoring should be emphasized, in line with current recommendations.<br>7- References: The reference list would benefit from revision and updating, as many citations were before 2020. In addition, more recent literature, including studies from the authors' own country, would strengthen the manuscript.<br>I hope these comments will be helpful in further improving the quality and clarity of the manuscript.

VERSION 1 – AUTHOR RESPONSE

Reviewer
Comment 1:
As the study population consists exclusively of children diagnosed with isolated language disorder, the title should more clearly reflect ILD specifically. In addition, delayed diagnosis is reported as a predictor; but cannot be an predictor, rather be an associated factor, therefore, the wording of the title may need to be revised.

Response:
We thank the reviewer for this important suggestion. In response, we revised the title to better reflect both the study population and the nature of the relationships examined. The title has been changed to: "Factors associated with isolated language disorder and special educational needs within the isolated language disorder group: a retrospective cohort study."

Comment 2:
While the authors report diagnosing isolated language disorder, the policy implications of this study extend beyond language assessment alone. At the health system and policy level, the findings underscore the importance of comprehensive developmental evaluation, as focusing solely on language may overlook other developmental vulnerabilities that influence outcomes and service needs.

Response:
We appreciate this valuable comment. In accordance with the reviewer's recommendation, we revised the section "How this study might affect research, practice or policy" to emphasize not only developmental screening, but also the importance of comprehensive developmental evaluation and ongoing developmental monitoring in children presenting with speech delay.

Comment 3:
The introduction would benefit from a more comprehensive background on ILD, including current knowledge and clinical considerations, before moving directly to the study aim.

Response: Thank you for this helpful suggestion. We expanded the Introduction to provide a broader background on isolated language disorder, including the heterogeneity of terminology in the literature and the rationale for our use of the term "isolated language disorder" in the present study.

Comment 4:
The finding regarding vitamin D deficiency appears to be based on retrospective file data, and the number of available biological markers is very limited, which restricts meaningful analysis. Given this limitation, I would suggest that the manuscript focus more strongly on clinically actionable factors such as early diagnosis, early intervention, and reduction of excessive screen time. Consideration

could be given to omitting vitamin D and other biological markers from the current manuscript. Prospective studies or randomized controlled trials would be needed for the role of vitamin D in children with ILD.

Response:
We thank the reviewer for this thoughtful comment. We agree that the vitamin D finding should be interpreted cautiously. Accordingly, we revised the manuscript to avoid implying a direct or causal relationship between vitamin D deficiency and isolated language disorder. We also rebalanced the Discussion, policy implications, and Conclusions to place greater emphasis on clinically actionable factors, including earlier identification, comprehensive developmental evaluation, ongoing monitoring, and reduction of excessive screen exposure. At the same time, we retained the vitamin D finding because it remained significant in the multivariable analysis; however, it is now presented more cautiously as an association requiring confirmation in larger prospective longitudinal studies.

Comment 5:
The term “delayed diagnosis” would be more accurate than “delayed presentation.” It is well established that delayed diagnosis of ILD is associated with poorer outcomes and an increased need for special education services.

Response:
We appreciate this important point. However, given the design of our study, we considered “later age at presentation” to be the more accurate term. Specifically, the interval between symptom onset and diagnosis could not be reliably determined from our retrospective data; therefore, we were unable to distinguish whether any delay was related to later help-seeking, delayed referral, or delayed diagnostic recognition. As the parameter available for analysis was the age at hospital presentation, we revised the wording accordingly and used “later age at presentation” throughout the manuscript. We believe that this term more precisely reflects the variable actually measured in our study. Nevertheless, we would be grateful for your reconsideration of this point and would be happy to adopt an alternative wording if you feel that it would be more appropriate.

Comment 6:
In addition to screening, the importance of ongoing developmental monitoring should be emphasized, in line with current recommendations.

Response:
Thank you for this suggestion. We revised both the “How this study might affect research, practice or policy” section and the Discussion to emphasize that, in addition to screening, children presenting with speech delay should undergo ongoing developmental monitoring and, when indicated, comprehensive developmental evaluation.

Comment 7:
The reference list would benefit from revision and updating, as many citations were before 2020. In addition, more recent literature, including studies from the authors’ own country, would strengthen the manuscript.
Response:
We thank the reviewer for this recommendation. The reference list has been revised and updated to include more recent literature, including relevant studies from our own country.

VERSION 2 – REVIEW

**REVIEWER NAME**	pekcici, Bahar Bingöler
**REVIEWER AFFILIATION**	Ankara University, Department of Developmental Pediatrics

**REVIEWER CONFLICT OF INTEREST**	No Conflict of Interest
**AI DISCLOSURE**	No, I have not used AI
**DATE REVIEW RETURNED**	14-May-2026

**GENERAL COMMENTS**	Thank you very much for the authors' responses to my comments.

**REVIEWER NAME**	Ttofari, Kerry
**REVIEWER AFFILIATION**	
**REVIEWER CONFLICT OF INTEREST**	No Conflict of Interest
**AI DISCLOSURE**	No, I have not used AI
**DATE REVIEW RETURNED**	27-May-2026

**GENERAL COMMENTS**	I have a number of recommendations for the authors to consider in a revision of the paper:<br><br>TITLE<br>Please revise. The consensus term is currently “Developmental Language Disorder” (DLD), however, the preference would be to use a term such as “language difficulties” for this paper because the children’s ages ranged from 9 months until 71 months. Furthermore, the assessments used included screening tests. The term DLD is usually not given until 5 years of age. Refer to: “Language can change a lot in the first 5 years of life. Some children who have difficulties at 2 might be doing well by 5, while others may still need support. This means speech pathologists may not officially diagnose DLD before 5 years of age. Speech pathologists might use terms like language difficulties, language delay or provide a provisional diagnosis of DLD.” (Developmental Language Disorder (DLD) **|** The DLD Project)<br><br>FIGURE 1<br>Please change “Lost of follow up” to “Loss at follow up”. Who made the recommendations of ‘watchful waiting’ and ‘special education’?<br>Introduction<br>• Although prevalence varies between studies, the figure most often quoted is one in 14 people. Please review this evidence brief with up to date references on DLD. Evidence Brief - Developmental Language Disorder (DLD) – October 2022. It would be preferable to refer to a general prevalence, rather than prevalence of 2 year olds, since children in your study were not all 2 year olds.<br>• I agree with the previous reviewer that more up to date references are required. Please see the above Evidence Brief for further references.<br>• First paragraph: change the second sentence to say “They are clinically characterised by difficulties in receptive and/or expressive language in any linguistic domain (morphology, syntax, semantics, pragmatics), for example, limited vocabulary and impaired sentence structure.<br>• There is no mention of speech-language therapists when tailored intervention strategies are mentioned (p. 8 of 48, line 59). It is important that readers are aware that there are speech and language therapists in Turkey and that children with language disorders should be referred to them for management (assessment, diagnosis and intervention), as they are experts in communication disorders. At this point you could consider also mentioning the

	importance of working in an interprofessional team.<br>• P. 9 of 48, lines 15-17: "concomitant receptive language impairment" and "limited expressive vocabulary" are part of DLD, rather than concomitant. They are part of the possible characteristics/ presentations of DLD.<br><br>METHODS<br>• Please clarify how all the variables were collected. Were they all based on parent report except for the Denver II, Bayley-III, laboratory investigations and recommended intervention types?<br>• P. 10 line 5: add "recommended" before "intervention types". Please note that although kindergarten attendance is listed here as a recommendation, it is not in Figure 1.<br>• It is unclear who completed the assessments. What were the qualifications of the child development specialists? Please provide more detail. Was it pediatricians, speech pathologists, occupational therapists, etc.?<br>• It is important that speech-language therapists are involved when a diagnosis of DLD and SSD is made. P. 10 of 48 lines 36-39: Who determined whether there were phonological errors? The most appropriate professional to make this diagnosis is a speech language therapist. Speech sound disorders include phonological errors AND articulation errors.<br>• The Ages and Stages Questionnaire was used for second tier evaluation, however, it is a screening tool (p. 10 of 48).<br>• I am curious whether there are any standardised language assessments used in Turkey for the diagnosis of DLD.<br>• P. 11 of 48 lines 46-53: I note that "Variables with <0.05 in univariable analyses were entered into a multivariable logistic regression model…", however, this did not seem to be the case. It appears that selected variables were entered into the regression model. Statistically significant variables of birth order, bilingualism and kindergarten attendance were not entered into the regression model. Please provide a rationale for this. You mention this in the discussion also (p. 16 of 48) but do not provide a rationale for why these were not included.<br>• Was the dependent variable in the logistic regression dichotomous? Or are there three outcomes: watchful waiting/ recommended special education during follow up/ recommended special education at initial presentation? This was unclear.<br>• When was the follow up? This was unclear. What was the time interval between the initial assessment and follow up? How many data points of collection were there? Please clarify this.<br>• P. 13 of 48: you mentioned you checked the association between vitamin D exposure and screen time. Can you please report the correlation coefficient? I agree with a previous reviewer about interpreting the Vitamin D results cautiously as this is a retrospective study.<br>• What were the criteria for special education at follow up? Was another assessment completed at this point? Was it based on paediatrician evaluation?<br>• What do you mean by special education services, within the context of Turkey? Please provide more information. Does this include specialised schools and/or services provided within mainstream schools? Does it include receiving allied health services (speech pathology, occupational therapy) and special education teaching?<br><br>DISCUSSION<br>• P. 15 of 48 line 25: please delete the word "aetiological".

	• P. 15 of 48, line 46 to 49: when you mention "comprehensive developmental evaluation", please include the importance of interprofessional management of children with DLD, including referral to a speech and language therapist for a comprehensive communication assessment (speech and language) and provision of intervention.<br>• Is one of the reasons for the age of presentation being significant related to the earlier age of presentation identifying "late talkers" who went on to have normal language development, whereas children presenting later had persistent communication difficulties?<br>• P. 17 of 48, line 31: do you mean "speech and language concerns" or just "speech concerns"? The same applies to p. 18 line 18.<br>• P. 18 of 48, line 20: change the term 'risk factors'.<br><br>I wish you well in your research. It would be great to see collaborations between medical professionals and speech language therapists from Turkish universities in future research relating to communication disorders.

VERSION 2 – AUTHOR RESPONSE

Reviewer 2

Comment 1:
TITLE
Please revise. The consensus term is currently "Developmental Language Disorder" (DLD), however, the preference would be to use a term such as "language difficulties" for this paper because the children's ages ranged from 9 months until 71 months. Furthermore, the assessments used included screening tests. The term DLD is usually not given until 5 years of age. Refer to: "Language can change a lot in the first 5 years of life. Some children who have difficulties at 2 might be doing well by 5, while others may still need support. This means speech pathologists may not officially diagnose DLD before 5 years of age. Speech pathologists might use terms like language difficulties, language delay or provide a provisional diagnosis of DLD." (Developmental Language Disorder (DLD) | The DLD Project)

Response: We fully agree with the reviewer's rationale regarding the developmental age span of our cohort. Because a substantial portion of our patient group is under 5 years of age, we have changed the core terminology from "isolated language disorder" to "language difficulties" throughout the manuscript, including the title, running head, abstract, main text, and tables.

Comment 2:
FIGURE 1
Please change "Lost of follow up" to "Loss at follow up". Who made the recommendations of 'watchful waiting' and 'special education'?
Response: The typographical error in Figure 1 has been corrected to "Loss at follow-up." We have also clarified the clinical decision-making workflow in the Methods section:
"Intervention recommendations were determined following a joint evaluation by a developmental pediatrician, a child development specialist, and a clinical psychologist. Recommendations for special education were made based on longitudinal monitoring of the child's language development. In addition, children with speech disorders or phonological errors, as well as some children with language difficulties requiring a special education report, were evaluated in collaboration with the Department of Speech and Language Therapy."

Comment 3:

Introduction
• Although prevalence varies between studies, the figure most often quoted is one in 14 people. Please review this evidence brief with up to date references on DLD. Evidence Brief - Developmental Language Disorder (DLD) – October 2022. It would be preferable to refer to a general prevalence, rather than prevalence of 2 year olds, since children in your study were not all 2 year olds.
• I agree with the previous reviewer that more up to date references are required. Please see the above Evidence Brief for further references.
• First paragraph: change the second sentence to say "They are clinically characterised by difficulties in receptive and/or expressive language in any linguistic domain (morphology, syntax, semantics, pragmatics), for example, limited vocabulary and impaired sentence structure.
• There is no mention of speech-language therapists when tailored intervention strategies are mentioned (p. 8 of 48, line 59). It is important that readers are aware that there are speech and language therapists in Turkey and that children with language disorders should be referred to them for management (assessment, diagnosis and intervention), as they are experts in communication disorders. At this point you could consider also mentioning the importance of working in an interprofessional team.
• P. 9 of 48, lines 15-17: "concomitant receptive language impairment" and "limited expressive vocabulary" are part of DLD, rather than concomitant. They are part of the possible characteristics/ presentations of DLD.

Response: We have updated the overall prevalence figures (1 in 14) and integrated the suggested 2022 DLD Evidence Brief citations into the Introduction.
The definition in the first paragraph has been revised exactly as recommended: "They are clinically characterized by difficulties in receptive and/or expressive language in any linguistic domain (morphology, syntax, semantics, pragmatics), for example, limited vocabulary and impaired sentence structure."
We also explicitly highlighted the crucial role of Speech-Language Therapists (SLTs) in Türkiye and the necessity of interprofessional management:
"In tailoring intervention strategies, working within an interprofessional multidisciplinary team—including speech-language therapists, developmental pediatricians, child development specialists, psychologists, and psychiatrists—is paramount." Finally, the phrasing implying that receptive impairments are "concomitant" to DLD has been removed.

Comment 4:
METHODS
• Please clarify how all the variables were collected. Were they all based on parent report except for the Denver II, Bayley-III, laboratory investigations and recommended intervention types?
• P. 10 line 5: add "recommended" before "intervention types". Please note that although kindergarten attendance is listed here as a recommendation, it is not in Figure 1.
• It is unclear who completed the assessments. What were the qualifications of the child development specialists? Please provide more detail. Was it pediatricians, speech pathologists, occupational therapists, etc.?
• It is important that speech-language therapists are involved when a diagnosis of DLD and SSD is made. P. 10 of 48 lines 36-39: Who determined whether there were phonological errors? The most appropriate professional to make this diagnosis is a speech language therapist. Speech sound disorders include phonological errors AND articulation errors.
• The Ages and Stages Questionnaire was used for second tier evaluation, however, it is a screening tool (p. 10 of 48).
• I am curious whether there are any standardised language assessments used in Turkey for the diagnosis of DLD.
• P. 11 of 48 lines 46-53: I note that "Variables with <0.05 in univariable analyses were entered into a

multivariable logistic regression model…", however, this did not seem to be the case. It appears that selected variables were entered into the regression model. Statistically significant variables of birth order, bilingualism and kindergarten attendance were not entered into the regression model. Please provide a rationale for this. You mention this in the discussion also (p. 16 of 48) but do not provide a rationale for why these were not included.
• Was the dependent variable in the logistic regression dichotomous? Or are there three outcomes: watchful waiting/ recommended special education during follow up/ recommended special education at initial presentation? This was unclear.
• When was the follow up? This was unclear. What was the time interval between the initial assessment and follow up? How many data points of collection were there? Please clarify this.
• P. 13 of 48: you mentioned you checked the association between vitamin D exposure and screen time. Can you please report the correlation coefficient? I agree with a previous reviewer about interpreting the Vitamin D results cautiously as this is a retrospective study.
• What were the criteria for special education at follow up? Was another assessment completed at this point? Was it based on paediatrician evaluation?
• What do you mean by special education services, within the context of Turkey? Please provide more information. Does this include specialised schools and/or services provided within mainstream schools? Does it include receiving allied health services (speech pathology, occupational therapy) and special education teaching?

Response: We appreciate the opportunity to make our methodological protocol fully transparent. We have addressed each point in the revised Methods section as follows:
1. Data Collection: The text has been updated to clarify that developmental variables were collected via structured developmental anamnesis interviews conducted with the primary caregivers, alongside standardized testing and electronic laboratory records.
2. Intervention Terminology: The word "recommended" was added before intervention types. While we initially aimed to categorize language enrichment counselling and kindergarten recommendations as two separate tiers, doing so resulted in underpowered, small subgroup sample sizes. To preserve statistical power when comparing children who required special education, we merged the kindergarten group into the broad "language development recommendations" group.
3. Assessors & Qualifications: Standardized developmental tests (Bayley-III and Denver-II) were administered by Child Development Specialists who hold Master's degrees in Child Development and are certified in the administration of standardized developmental tests.
4. Role of Speech-Language Therapists: We agree that an SLT is the gold-standard professional for diagnosing DLD, SSD, and phonological errors. In our state university hospital framework, initial developmental triage is performed by developmental pediatricians; but all patients with speech disorders and phonological errors, as well as some patients with language disorders requiring a special education report, were evaluated in collaboration with the Department of Speech and Language Therapy.
5. Screening Tools: The classification of the ASQ has been corrected to a screening tool.
6. Standardized Language Tests in Türkiye: Standardized language-specific assessments are available in Türkiye, including TİFALDİ, TEDİL, and the Ankara Articulation Test, and are typically administered by Speech and Language Therapists. However, because our study was retrospective and these test results were not available for all patients, they could not be incorporated into the present analysis.
7. Regression Variable Selection Rationale: We added the explicit statistical rationale for the excluded univariable parameters:
"Kindergarten attendance lost its statistical significance when adjusted for the 36-month institutional age threshold in our country ($p = 0.6$) and was excluded to prevent confounding bias. Bilingualism was excluded due to an exceedingly low cell count (n=9 total), which would introduce sparse data bias into the regression model. Lastly, birth order and the total number of siblings demonstrated high collinearity; to avoid multicollinearity, only 'number of siblings' (which possessed the stronger

univariable p-value) was retained in the final multivariable model."
8. Dependent Variable & Follow-up: The primary multivariable logistic regression utilized a dichotomous dependent variable (Language Difficulties vs. Typically Developing). Following this, a secondary longitudinal subgroup analysis was performed within the Language Difficulties cohort to evaluate Special Educational Needs (SEN). Follow-up assessments occurred at routine 3-month intervals. We have incorporated the follow-up timing to special education recommendation into the text, reported correctly as a median of 3.0 (IQR: 1.0–6.0) months.
9. Vitamin D vs. Screen Time Correlation: When evaluating the relationship between Vitamin D deficiency (<20 ng/mL) and daily screen time (>2 hours/day), a chi-square test of independence revealed no statistically significant correlation ($\chi^2(1)=1.196$, $p=0.274$)
10. Special Education Context in Türkiye: We added contextual details regarding our national system: "In Türkiye, upon receiving a formal Special Education Board Report from a tertiary hospital, state social security covers 2 to 3 hours of individualized therapy per week. This education is delivered by allied health professionals (primarily SLTs) at private Special Education and Rehabilitation Centers audited by the Ministry of Education."

Comment 5:

DISCUSSION

• P. 15 of 48 line 25: please delete the word “aetiological”.
• P. 15 of 48, line 46 to 49: when you mention “comprehensive developmental evaluation”, please include the importance of interprofessional management of children with DLD, including referral to a speech and language therapist for a comprehensive communication assessment (speech and language) and provision of intervention.
• Is one of the reasons for the age of presentation being significant related to the earlier age of presentation identifying “late talkers” who went on to have normal language development, whereas children presenting later had persistent communication difficulties?
• P. 17 of 48, line 31: do you mean “speech and language concerns” or just “speech concerns”? The same applies to p. 18 line 18.
• P. 18 of 48, line 20: change the term ‘risk factors’.

Response: The word "aetiological" has been deleted.

We inserted the recommended emphasis on interprofessional SLT referral into the Discussion: "Due to the diagnostic complexity, interprofessional collaborative management involving Speech-Language Therapists, child psychiatrists, and pediatric neurologists is essential for detailed communication assessments and targeted intervention."

We agree with the reviewer that this finding may, at least in part, reflect the inclusion of children with a transient “late talker” trajectory within our broader language difficulties group. We have therefore revised the Discussion to acknowledge this possible explanation more explicitly.

"Speech concerns" was corrected to "speech and language concerns" throughout.

The phrase "risk factors" was updated to "associated factors".

Comment 6: I wish you well in your research. It would be great to see collaborations between medical professionals and speech language therapists from Turkish universities in future research relating to communication disorders.

Response: We value this recommendation and agree that closer collaboration between medical professionals and Speech and Language Therapists will strengthen future research in communication disorders. At our university, we already work in collaboration with the Department of Speech and Language Therapy, and we believe that further integration of SLTs into our clinical team would strengthen both clinical care and research.
